# Orientation-specific learning of the prior assumption for 3D slant perception

**DOI:** 10.1038/s41598-018-29361-2

**Published:** 2018-07-23

**Authors:** Shuichiro Taya, Masayuki Sato

**Affiliations:** 1 Keio University, 4-1-1, Hiyoshi, Kohoku-ku, Yokohama 223-8521 Japan; 2 The University of Kitakyushu, 1-1, Hibikino, Wakamatsu-ku, Kitakyushu 808-0135 Japan

## Abstract

We usually interpret a trapezoidal image on our retina as a slanted rectangle rather than a frontoparallel trapezoid, because we use a statistical assumption (i.e. rectangles are more common than trapezoids), called a ‘prior’, for recovering the 3D world from ambiguous 2D images. Here we report that the shape prior for recovering 3D slant can be updated differently depending on the slant axis orientation (horizontal vs. vertical). The participants were exposed to a variety of trapezoidal images surrounded by a stereoscopic reference plane. The perspective transformation of the images was interpreted as 2D shape, rather than 3D slant because the surrounding plane enhanced disparity. We found that, after continuous exposure to such images, the participants relied less on the shape information for recovering 3D slant, suggesting the update of priors via experience (i.e., rectangles are *less* common than trapezoids). Importantly, the learning effect was context (slant-axis) specific although partially transferred across contexts; the training with horizontal-axis slant reduced the reliance on perspective even in vertical-axis slant estimation but not vice versa. The results suggest that context-specific training is vital to update the prior for the horizontal-axis slant, whereas it is not required to update the prior for a vertical-axis slant.

## Introduction

We can perceive the 3D structure of the external world even when it is represented in drawings and photographs where neither binocular disparity (slight image differences between the two eyes’ views) nor motion cues to depth (e.g. motion parallax) are available. This is because our visual system can utilise pictorial cues such as linear perspective, shading, and texture gradient to recover 3D structure from 2D images. To use these pictorial cues, however, we need to have assumptions about the statistical regularities in the visual environment (i.e. ‘priors’). For example, seeing depth from texture gradient relies on a prior that texture elements are homogeneous and/or isotropically distributed^1–3^. Interpretation of shapes from luminance gradients (shape-from-shading) relies on the ‘light-from-above’ prior^4–6^, while perceiving slant in depth from the figural foreshortening cue requires an ‘isotropic shape’ prior^7–9^.

Previous studies have revealed that internal statistics can be updated according to recently experienced stimulus statistics^8–12^. For example, the assumption of light source position for recovering 3D shape from shading cues can be modulated to match the direction suggested by haptic^10^ or visual feedback^11^. For the isotropic shape prior, the observers relied progressively less on figural foreshortening as a cue to slant in depth after exposure to a series of visual stimuli in which the aspect ratio of stimuli had high divergence^9^.

An intriguing question regarding learning priors is how much the learning depends on a certain category or context of stimuli. Evidence addressing this issue from previous studies is mixed. Seydell *et al*.^9^ reported that learning of an ‘isotropic shape’ prior for 3D slant could be specific to a particular shape category (circle vs. diamond), but not if it is associated with a particular colour category (pink vs. purple). Adams *et al*.^10^ suggested that the assumption of light source position is not specific to the stimulus shapes (circular dimple vs. trapezoidal ridge), whereas in a later study Kerrigan and Adams^12^ reported that the observers could learn different light source priors depending on the environment light colour. The mixed results can be interpreted by the ecological validity of changes in the statistics regarding the actual environment. For example, the learning of a light source prior was non-specific among different shapes^10^ because the changes in a light source direction inevitably alter shading pattern in the environment regardless of the object’s shape. On the other hand, it has been reported that the light source priors could be specific to different light colours^12^. This might be because lights from different directions could have different colours (e.g. white sunlight shining from above vs. a reddish campfire illuminating from the side). In a similar vein, learning of the isotropic shape prior was specific to particular shapes^9^, because the divergence of aspect ratios of the shape of an object could depend on the object categories; e.g., coins in most cases have perfectly circular shape whereas the aspect ratio of broaches would have wider variety^9^. On the other hand, the learning shape prior could not be colour specific because colour is not directly linked to the shape.

Taking into account the context specificity and non-specificity in the learning reported in the previous research, it would be intriguing to test whether the learning of prior knowledge about 3D slant is specific to the slant axis orientation. In our daily life, slant about a horizontal axis (H-axis slant) and slant about a vertical axis (V-axis slant) are often given in different contexts. The examples of H-axis slant are floors, table tops, etc. and the examples of V-axis slant are walls which are observed from aside. The question is whether we can possess and update independent priors for each direction of slant. Possessing context specific priors enable more veridical depth estimation but would delay adaptation to changes in the environmental statistics. Generalisation of priors across different contexts might have the benefit that fewer resources are required, but at the same time, it is less useful if the statistical regularities are considerably different among contexts (see Roach *et al.*^13^, for the related discussion). The goal of this study was to investigate which of these scenarios would be the case.

## Overview of Current Experiment

This study tested the learning of the ‘rectangular prior’, the internal statistical model about the stimulus shape that is required for recovering 3D slant from linear perspective cue^14^. Recovering 3D shape from linear perspective requires a prior assumption about the statistics of the stimulus shape. Specifically, our visual system uses the assumption that objects in the world are roughly rectangular; i.e. most quadrangular shapes consist of lines parallel or perpendicular to each other. Because of this assumption, we tend to interpret a trapezoidal-shaped retinal image as a projection of a rectangle slanted in depth, in spite of the fact that a frontoparallel trapezoid can produce the same retinal image.

In our experiment, changes in the internal statistics about stimulus shapes were assessed by measuring changes in the participant’s reliance on the perspective cue relative to binocular disparity. Toward this end, participants were presented stereograms of a slanted plane containing conflicts between disparity and perspective cues to slant. We measured participant’s cue weighting (i.e., relative reliance on perspective information vs. binocular disparity) before and after exposing them to an environment where stimulus shapes were mostly interpreted as trapezoids instead of rectangles. To introduce such a learning environment, we here utilised a stereoscopic reference plane that surrounds the ‘slanted’ central plane. It has been reported that the stereoscopic reference plane facilitates the binocular disparity cue to slant in depth^15–20^. For example, in Gillam *et al*.^15^, the perceived slant of a stereogram was almost exactly as specified by disparity when it was presented with a frontoparallel reference plane, even though monocular cues (i.e., linear perspective and texture on the surface) strongly suggested that the stimulus was parallel to the frontal plane (see Figs 2 and 3 in Gillam *et al*.^15^; see also van Ee^19^). The result suggests that when a stereoscopic reference plane is present, perspective information (i.e. converging lines) is less associated with stimulus slant. Rather, the converging lines would be attributed to the non-rectangular shape of the stimulus. That means, observers may perceive a frontoparallel trapezoid, instead of a rectangle slanted in depth when a trapezoidal image is projected on their retina. Through longitudinal exposure to such a ‘trapezoidal world’, observers may update their internal statistics about the shape regularity such that the environment has a larger population of trapezoids. This would result in an effective down-weighting of perspective cues.

The key issue of the present study is whether the learning of the ‘rectangular prior’ transfers between horizontal and vertical slant-axis orientations. If the learning can be context specific, its effect (down-weighting of the perspective cue) will not be observed when the learning was developed with one slant orientation and the post-learning weight is measured with the other orientation. On the one hand, the learning of one slant orientation can be transferred to any unlearned orientations. We tested this issue by comparing the strength of the learning effect between the intra-axis conditions where both the learning and the post-leaning measurement was made with the same (horizontal or vertical) slant-axis, and the inter-axis conditions where the learning and the post-learning measurement was made with orthogonal slant-axes.

## Methods

### Apparatus and stimuli

Stimuli were generated using a VSG 2/5 graphics card (Cambridge Research System) and presented on a 21-inch CRT monitor (Sony, GDM-F520) with a resolution of 1024 × 768 pixels and a refresh rate of 120 Hz. Anti-aliasing techniques were applied to achieve sub-pixel resolution. Participants observed the stimuli at a viewing distance of 57 cm. Head motion was restricted by a chin-rest.

**Figure 1:**
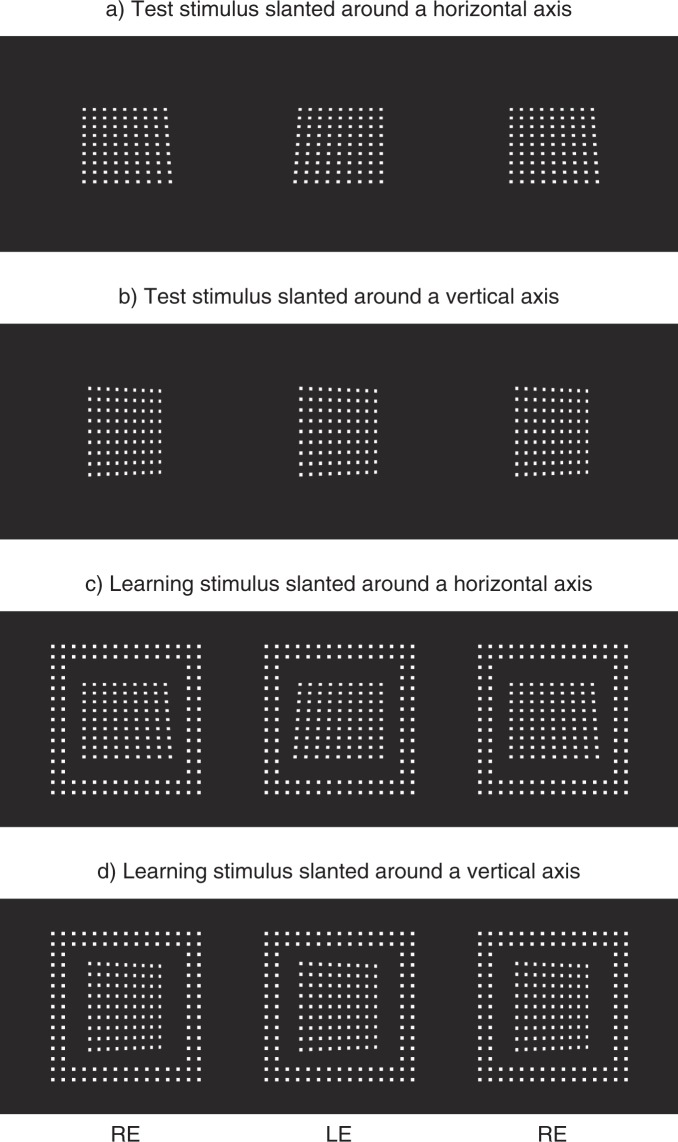
Stereogram used for the measurement of cue weighting. Disparity and perspective specify different slant angle in the central surface (**a**–**d**), while they concordantly specify 0° slant (frontoparallel) in the surrounding plane (**c**,**d**). Left and centre images are for cross fusion, and centre and right images are for parallel fusion.

The stereoscopic stimuli consisted of a regular grid-array of small white rectangles, which were presented against a black background (Fig. 1). The central, planar test surface was slanted around either a horizontal-axis (Fig. 1a,c) or a vertical-axis (Fig. 1b,d). This test surface subtended 7.5° × 7.5° in visual angle when it was parallel to the frontal plane. In the learning phase, a frontoparallel reference frame (12.9° × 12.9°; 1.2° width) was also presented around the test surface (Fig. 1c,d). Binocular disparity and linear perspective specified different slants in the test surface, whereas in the reference they always specified 0° slant (frontoparallel) concordantly.

The dichotic-half images of the stereogram were viewed through liquid-crystal shutter goggles (Stereographics, CrystalEyes3) that were synchronised with the monitor frame rate. To reduce cross-talk, the stimuli were drawn in red to take advantage of the faster red phosphor. Particular care was taken to make the laboratory in which the experiments were carried out completely dark, since for the present study it was critically important to prevent irrelevant objects, which can serve as a reference plane (especially the frame of the CRT display), being invisible to the participants. All data were collected in a dark chamber covered with blackout curtains. An orange transparent filter was set in front of the participant to prevent possible cross-talk. The filter passed the red light of the stimulus and reduced the residual light from the black background. The brightness of the stimulus and of the background measured through the goggles and filter was 1.8 cd/m^2^ and 0.0 cd/m^2^, respectively.

### Procedure

We used the weighted linear combination rule, which is widely used to describe perceived slant specified by stereo and monocular cues^19–24^, to evaluate the participants’ cue combination strategy:

1 $$\hat{S}={w}_{p}{S}_{p}+{w}_{d}{S}_{d}$$

Equation 1 indicates that perceived slant $(\hat{S})$ is determined by a combination of perspective-specified slant (*S*_*p*_) and disparity-specified slant (*S*_*d*_), each weighted by coefficients *w*_*p*_ and *w*_*d*_, respectively (*w*_*p*_ + *w*_*d*_ = 1).

The weights assigned to perspective and disparity were measured in five separate sessions, each of which was conducted on a separate day. Each session consisted of three phases: pre-training test, training, and post-training. The changes in cue weighting (i.e., the learning effects) were evaluated by comparing the cue weighting before and after the training phase. Each session was conducted once per day, and there was a break of at least one day between the sessions.

Two intra-axis learning conditions (in which the training and tests were conducted with the same slant-axis) and two inter-axis learning conditions (in which the training and tests were conducted with orthogonal slant-axes) were used to assess the interaction between the training slant-axis and the test slant-axis, namely HH (training and test were conducted with H-axis slant), HV (training with H-axis slant and test with V-axis slant), VH (training with V-axis slant and test with H-axis slant), and VV (training and test with V-axis slant). Eight participants completed the horizontal-axis training conditions (HH and HV), whilst the others completed the vertical-axis training conditions (VH and VV).

Cue-weighting was assessed with the ‘slant nulling’ method, which is a similar procedure to that used in a previous study^21,25^. In the training and the test phases, participants were asked to adjust the slant of the central test surface by a pressing a button until it appeared parallel to the frontoparallel plane. Each press of the button changed the simulated slant angle of the central surface specified by disparity as well as that specified by perspective by 1° in the same direction. The stimulus was visible until participants finished the slant adjustment.

In the pre-training and post-training test phases, the initial angle of *S*_*d*_ and *S*_*p*_ were defined by the following equations:

2 $$\begin{array}{ccc}{S}_{d} & = & {S}_{r}\pm \alpha ;\\ {S}_{p} & = & {S}_{r}\mp \alpha \end{array}$$

*S*_*r*_ was determined randomly from a range of −30° to +30° (with a step size of 1°, the positive value means top-far or right-far slant) in each trial. The value of *α* was either −15° or +15°, so that the angle of slant specified by disparity and that specified by perspective were consistently different by 30°. Each stimulus was presented eight times in a blocked random order. Participants completed 16 trials in each test phase, namely two directions of slant axes (horizontal- and vertical-axis slant) × 2 values of *α* (−15° and +15°) × 4 repetitions.

In the learning phase, the initial values of *S*_*d*_ and *S*_*p*_ were again defined as shown in Equation 2. For learning, *S*_*r*_ was selected randomly from the range −15° to +15°, and *α* was selected from 0°, 7.5°, 15°, 22.5°, or 30°. Therefore, the conflict between *S*_*d*_ and *S*_*p*_ was 0°, 15°, 30°, 45°, or 60°. Each stimulus was presented twice in a blocked random order and participants completed 18 trials in each training phase, consisting of one slant axis (horizontal- or vertical-axis slant) × 9 values of *α* (−30°, −22.5°, −15°, −7.5°, 0°, +7.5°, +15°, +22.5°, +30°) × 2 repetitions for each.

### Participants

Twenty-five volunteers participated in this study. All of them had less than 30 arcsec stereo thresholds (range: 5.6–27.6 arcsec; median: 7.9 arcsec) as measured with a stereo test similar to Coutant and Westheimer^26^. All were naïve as to the experimental purpose. Eight participants were assigned to the training condition with H-axis slant (H-axis training condition) whereas the remaining participants were assigned to the training condition with V-axis slant (V-axis training condition). It is well known that there is a marked anisotropy in disparity-defined slant perception; i.e. slants around a vertical axis show a higher detection threshold, slower fusion latency, and smaller perceived slant than slants around a horizontal axis^15,16,27–29^. Therefore, we could predict that not a few participants would assign a considerably lower weight for binocular disparity and thus would see the slant from this perspective even in the training phase. In theory, we could not expect the learning effect with such participants, because they would see slanted rectangles rather than frontoparallel trapezoids during the training phase. If this type of participant made up the majority of the participants under the V-axis training condition, we could not expect to obtain learning effects under this condition. In this case, it would be hard to distinguish that the absence of learning was due to the nature of the stimulus itself (i.e. learning could not be established with V-axis slant training after all) or due to a lack of experience (i.e. no prolonged exposure to an environment where trapezoids are more common than rectangles), which is essential to update the internal statistics. Because we could not predict how many participants would have difficulty seeing the disparity-defined slant around a vertical axis (and it was not sure that this kind of participant would not show a learning effect), we assigned the greater number of participants to the V-axis training condition.

Participants were given informed consent and the ethics committee of the University of Kitakyushu approved the study. All experimental procedures were conducted in accordance with the guidelines and regulations of the ethics committee of the University of Kitakyushu.

### Data availability

The datasets generated during and/or analysed during the current study are available from the corresponding author on reasonable request.

## Results

**Figure 2:**
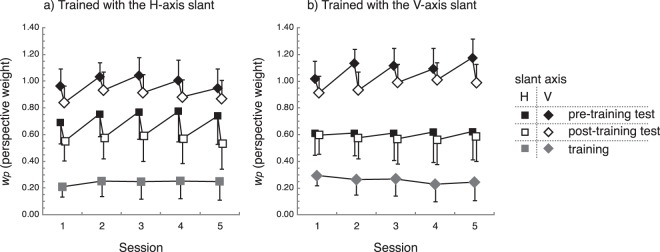
Average perspective weights as a function of session number. (**a**) Results from participant group who were trained with H-axis slant (N = 8). (**b**) Results from participant group who were trained with V-axis slant (N = 17). Weights measured with the H-axis slant are plotted with squares and weights measured with the V-axis slant stimuli are plotted with diamonds. Black symbols, white symbols, and grey symbols are the results of the pre-test training phase, post-test training phase, and training phase, respectively. Error bars denote one standard error of the mean (SEM).

Figure 2 shows the group mean of perspective weights plotted as a function of the number of experiment session. Three important findings can be drawn from this figure. First, the presence of the surrounding reference plane conspicuously reduced the perspective weight in both the orientations of slant axes (see grey symbols). The down weighting of perspective denotes that the participants attributed the trapezoidal shape of the stimulus contour more to the shape of the object, rather than the perspective projection caused by the slant in depth. In other words, in the training phase (where the reference plane was always presented) the experience of the participants was that a larger population of quadrangular objects in the environment are trapezoids, not rectangles. Second, in accordance with our expectation, perspective weight was reduced after the training phase in the all conditions, except the VH condition (square symbols in Fig. 2 right panel). Third, the learning effects were not persistent; the cue weighting of the participants returned to around the value of initial measurement after at least a 24-hour interval. A statistical analysis supported the second and the third points. The repeated-measures two-way analysis of variance (ANOVA) with the factor of training (pre-training/post-training) and sessions (1–5) was conducted on the measured perspective weights separately for each of the four experimental conditions (HH, HV, VV, and VH). This revealed that the significant main effects of training in the HH, HV, and VV conditions (HH: *F*_1,7_ = 31.45, *p* = 0.00, η^2^ = 0.03; HV: *F*_1,7_ = 48.01, *p* = 0.00, η^2^ = 0.02; VV: *F*_1,16_ = 10.90, *p* = 0.01, η^2^ = 0.07) but not in the VH condition (*F*_1,16_ = 0.70, *p* = 0.42, η^2^ = 0.00). The main effects of sessions and the interaction were not significant in all of the four conditions (all *p* > 0.05), indicating the absence of carryover of the learning effect. Hence, the effects of sessions were not significant, we used the data averaged across sessions in the later analyses.

**Figure 3:**
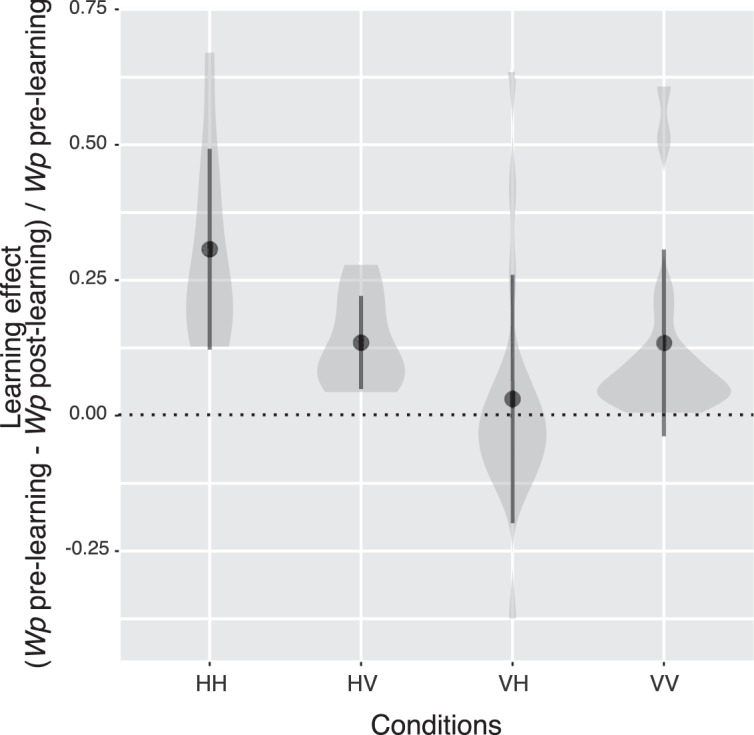
Violin plots for the learning effect obtained from the four experimental conditions. Filled circles denote the group mean. Error bars represent ±1 SD.

Figure 3 plots the group mean of learning effects separately for the conditions. Here the learning effects were calculated as the ratio of the changes in *w*_*p*_ before and after the training $(\frac{{w}_{p}pre-{w}_{p}post}{{w}_{p}pre})$. The analysis again suggests the absence of learning transfer in the vertical-axis training condition. One-sample two-tailed *t*-tests with a Bonferroni correction revealed that the learning effects were significantly larger than zero in the HH, HV, and VV conditions (*t*_7_ = 4.69, *p* = 0.01, *d* = 1.66; *t*_7_ = 4.44, *p* = 0.01, *d* = 0.78; *t*_16_ = 3.21, *p* = 0.02, *d* = 1.57, respectively) but not in the VH condition (*t*_16_ = 0.55, *p* = 2.35, *d* = 0.13). We also conducted 2 × 2 (training axis vs. test axis) mixed design ANOVA on the learning effect. The main effect of training axis was significant, indicating that the training with H-axis slant produced a larger learning effect than the training with V-axis slant (*F*_1,23_ = 4.65, *p* = 0.04, η^2^ = 0.10). The main effect of test axis was not significant (*F*_1,23_ = 0.52, *p* = 0.48, η^2^ = 0.01). There was a significant interaction between the training axis and test axis (*F*_1,23_ = 8.29, *p* = 0.01, η^2^ = 0.10). The following simple main effect analysis revealed that the learning effect was stronger in the HH condition than the HV condition (*F*_1,7_ = 7.87, *p* = 0.03, η^2^ = 0.29), but the difference in the learning effect between the VH condition and the VV condition was not significant (*F*_1,16_ = 3.10, *p* = 0.10, η^2^ = 0.07).

**Figure 4:**
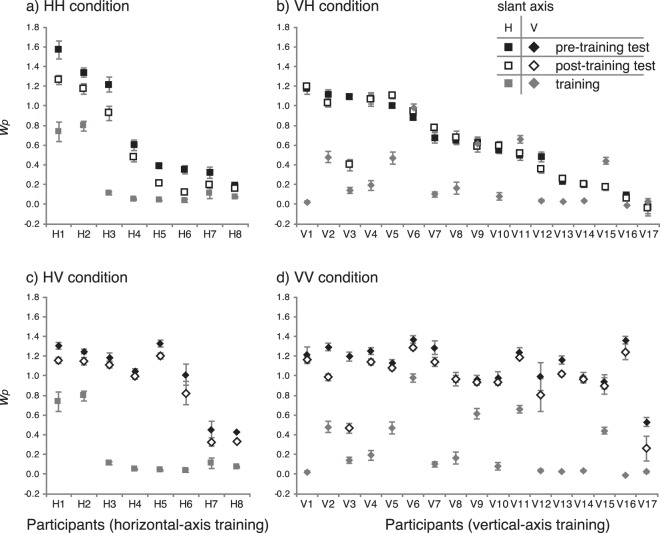
Perspective weights for individual participants in the pre-training test phase (black symbols), training phase (grey symbols), and post-training test phase (white symbols). The panels show the result of the (**a**) HH, (**b**) VH, (**c**) HV, and (**d**) VV conditions. Perspective weights measured with the H-axis slant stimuli are plotted with squares and those that were measured with the V-axis slant stimuli are plotted with diamonds. Error bars represent ± 1 SEM.

Individual differences in slant-cue weighting have repeatedly been reported^8,22,25^. Our participants also showed a wide range of individual differences in cue weighting (Fig. 4). Note that, in five participants in the VH condition (Fig. 4b, V6, V9, V11, V15, and V17) the *w*_*p*_ was larger in the training phase than in the pre-training test phase. In theory, we should not expect changes in the internal statistics of those participants, since the statistical distribution of stimulus shapes they would estimate in the training might not be different from that in the pre-training (in other words, they saw slanted rectangles rather than frontoparallel trapezoids even during the training). One might think those participants are responsible for the absence of significant learning in the VH condition. However, the learning effect was not significantly different from zero even after excluding those five outliers (*p* = 0.16 with no correction). Thus, it is not reasonable to attribute the null learning effect in the VH condition to these outliers.

## Discussion

Our results included two novel implications about the learning of internal statistics (priors) for depth perception. First, the interaction among depth cues can play an important role for updating priors. In our training phase, participants experienced the “trapezoidal world” induced by the stereoscopic reference frame, indicating that stereoscopic context (e.g., reference frame) could constrain the interpretation of monocular image cue and this constraint could encourage to them to re-interpret the assumption about the statistical regularity of shapes in an environment. Second, our visual system can separately update different priors depending on the stimulus orientation: The learning effect (reduction of reliance on the perspective cue) was larger when it was measured with the slant orientation that was the same as that of the learning period. This is the first report of spatial orientation serving as a context for the priors for depth perception. However, the reduction of perspective cue was observed not only for the slant orientation exposed during the training but also for the slant orientation orthogonal to that (i.e. the HV condition). Thus, the learning was partially transferred across contexts and not completely context-specific. In addition, no such ‘transfer’ of learning was obtained when the participants were trained with the vertical-axis slant (i.e. the VH condition), a point we return to later in this discussion.

### Learning of internal statistics

The data from the intra-axis conditions (HH and VV) clearly show that the participants reduced the weight of perspective for the estimation of 3D slant after exposure to the stimuli that suggested that the environment contains a large proportion of trapezoids. We suggest that the changes in cue-weighting observed here were derived from changes in the participant’s assumption about the relative proportion of rectangles and trapezoids in the world. The significant reduction of perspective weights in the training phase relative to their pre-training level (see grey symbols in Fig. 2) indicates that the participants interpreted that a trapezoidal image on the retina was not the perspective projection of slanted rectangle, but the stimulus shape; in other words, the stimulus is not a slanted rectangle but a frontoparallel trapezoid. By prolonged exposure to such stimuli, participants updated their internal models about stimulus statistics in such a way that the quadrangular objects in the present environment mostly consisted of trapezoids. As a result, the relative reliability of perspective transformation (of stimulus image) as a depth cue is reduced. Our results suggest that, in line with previous studies^8–12^, the visual system can update the internal statistical model on the basis of experience. Furthermore, our results showed that the stereoscopic cue can be influential for establishing statistical learning for pictorial depth cues.

An alternative explanation of the present results might be given in terms of cue-conflict based recalibration^25,30–33^. It has been suggested that when two cues signal different depths, like in the stereograms used in the present study, the visual system ‘recalibrates’ the association between a cue value and perceived depth in a direction that compensates for the cue conflict. Thus, one might argue that, in the case of present study, the disparity-specified slant and the perspective-specified slant might be calibrated to resolve the conflict between them, producing the apparent changes in cue weighting reported here. However, this scenario is not applicable to the current results because in our experimental setting the conflicts between disparity-specified slant and the perspective-specified slant were bidirectional. That is, even if the calibration had taken place in one learning trial in which *S*_*p*_ = *S*_*d*_ + α, for example, the effect of the calibration would have been cancelled out by the calibration taking place in the trial with *S*_*p*_ = *S*_*d*_ − α. In such way, the operation of calibration could not lead the changes in cue weighting.

### Context specificity and non-specificity of learning

The learning effect (the reduction of *w*_*p*_, relative reliance on the perspective cue) was larger in the intra-axis conditions (HH and VV) than in the inter-axis conditions (HV and VH). These results suggest that the learning of rectangular priors is, at least to some extent, context-specific; i.e., we can develop different priors for different contexts. However, after the training with H-axis slant, *w*_*p*_ was significantly reduced not only in the H-axis slant but also in the V-axis slant. Thus, the learning was not strictly slant-axis specific. Similar partial inter-context transfer of learning has been reported with light priors for shape from shading^12^.

Unexpectedly, we found an asymmetry in the transfer of learning between the H-axis slant and the V-axis slant. Specifically, training with the H-axis slant led to the reduction of *w*_*p*_ in the V-axis slant as well, whereas training with the V-axis slant did not change the *w*_*p*_ in the H-axis slant. Based on the results of this study, at least the following conclusions can be drawn. First, training with the H-axis slant (i.e. exposing participants to the environment which has a larger population of short-side-left/right trapezoids exemplified in Fig. 1d) is vital to overwrite the prior used in recovering the H-axis slant. In other words, updating the prior for the H-axis slant is ‘orientation specific’. On the other hand, the prior used for recovering the V-axis slant could be updated independently of the orientation of the trapezoids presented during the training period. In other words, updating the prior for the V-axis slant is ‘orientation non-specific’. Alternatively, one might think that we can explain the absence of learning under the VH condition by assuming the existence of a specialized prior for the V-axis slant; i.e. training with the V-axis slant only updates the V-axis specific prior, and thus does not change the perception of the slants around the other axes. However, in this case, it is difficult to explain why not only the V-axis training but also the H-axis training can update the prior that should be V-axis specific (i.e. the HV condition).

Why is specialized training required to update the prior for the H-axis slant, whereas it is not required to update the prior for the V-axis slant? It is difficult to explain the reason using only the current results. Nevertheless, we can derive some hypothetical explanations from the suggestions of previous studies, and describing such explanations would be useful for future research. We show the speculative scenario below. The results probably reflect the specificity of slant about a horizontal-axis as a ‘context’ of learning. In our environment, we often directly interact with the H-axis slant surface more than the V-axis slant surface; for example, the estimation of H-axis slant is important when we are going to walk on a hilly road, place a mug on a table top, etc. If we misestimate the degree of slant in this context we may waste our energy for walking up a hill, or may spill coffee by putting a mug on a slanted surface. Such opportunity of direct interactions is rare for the V-axis slant. In this way, the estimation of the H-axis slant could have an ecologically important meaning greater than the estimation of the V-axis slant. A recent study suggests that the direct interaction with the environment is a key element of the specialization of learning priors^13^. According to this study, we initially develop a single general prior, then when they are coupled with different motor outputs (action/behavior) multiple specific priors are formed. As mentioned above, we might have more opportunities for direct interactions with the environment for the H-axis slant. Thus, a prior that is specialized for the H-axis slant might have been formed through the daily life, whereas for the V-axis slant this possibility is low. If this is the case, direct experience of statistical changes about the H-axis slant must be required to update the prior which is specialized to this context. On the other hand, the estimation of the V-axis slant can be affected by statistical changes on slants in any directions, because, unlike the H-axis slant, the V-axis slant will not form the special prior.

## Conclusion

We examined whether learning the internal statistics regarding quadrangular shapes (‘rectangular prior’) is context specific or transferred across contexts. The results showed that training with the slant around a horizontal axis not only updated the prior for the trained (horizontal) axis slant, but also updated the prior for the untrained (vertical) axis slant. On the other hand, training with the slant around a vertical axis only updated the prior for the trained slant. We concluded that context-specific training is required to update the prior of the horizontal-axis slant, whereas it is not required to update the prior of the vertical-axis slant. Why a certain slant orientation requires context-specific training whereas the others do not when updating the shape prior remains an intriguing topic for future study.
